# MILP-based optimal day-ahead scheduling for a system-centric community energy management system supporting different types of homes and energy trading

**DOI:** 10.1038/s41598-022-22293-y

**Published:** 2022-10-31

**Authors:** Huy Truong Dinh, Dongwan Kim, Daehee Kim

**Affiliations:** 1 School of Computer Science & Engineering, The Saigon International University (SIU), Ho Chi Minh City, Vietnam; 2 Department of Electronics Engineering, Dong-A University, Busan, 49315 South Korea; 3 Department of Future Convergence Technology, Soonchunhyang University, Asan, 31538 South Korea

**Keywords:** Electrical and electronic engineering, Energy infrastructure

## Abstract

Optimal day-ahead scheduling for a system-centric community energy management system (CEMS) is proposed to provide economic benefits and user comfort of energy management at the community level. Our proposed community includes different types of homes and allows prosumers to trade energy locally using mid-market rate (MMR) pricing. A mathematical model of the community is constructed and the optimization problem of this model is transformed into an MILP problem that can be solved in a short time. By solving this MILP problem, the optimization of the overall energy cost of the community and satisfaction of the thermal comfort at every home are achieved. For comparison, we also establish two different CEMSs for the same community: a prosumer-centric CEMS and no CEMS. The simulation results demonstrate that the community with the proposed CEMS has the lowest daily energy cost among three CEMSs. In particular, the community with the proposed CEMS only has $78\%$ of the daily energy cost of the community with the prosumer-centric CEMS. Moreover, by using linear transformation, the computational time of the optimization problem of the proposed system-centric CEMS is only 118.2 s for a 500-home community, which is a short time for day-ahead scheduling of a community. We finally investigate the trade-off of the MMR pricing in the local energy trading of the community, which allows the profits of different types of homes to be flexibly adjusted.

## Introduction

With the rapid increase in many distributed energy resources (DERs), such as renewable energy systems (RESs) and energy storage systems (ESSs) at many smart homes, community energy management systems (CEMSs) for local energy communities (LECs) to reduce the energy cost of this group and increase the benefit for each member have received substantial research attention. A CEMS generally includes two parts: energy management for its DERs and energy trading for its members. The approaches of a CEMS can mainly be divided into two categories depending on the management of the DERs: the prosumer-centric and system-centric approaches^1^. The first category allows prosumers to maintain complete control of their DERs. However, the integration of the information of the trading model into the scheduling model of the prosumer devices at each home is a significant challenge. Hence, this approach is usually divided into two stages. In the first stage, each prosumer schedules their DERs to optimize the individual energy cost without information of the trading model. In the second stage, prosumers join the local market to improve their benefits^2–7^. The second category focuses on the construction of a central entity that collects the information of all DERs inside the community, and schedules them to optimize its overall objectives and maximally match the electricity demand of consumers with the electricity generation of producers within the community^8–10^.

There are mainly two types of DERs inside a LEC: individual DERs and communal DERs. Individual DERs set up by residents are integrated into a smart home and the residents of that home can fully control the individual DERs. Communal DERs or shared DERs are independent entities from smart homes and residents cannot control these communal DERs. For the LEC with communal DERs, a system-centric CEMS with an operator is usually needed to control and provide their energy to every home optimistically^11–15^.

The local energy market (LEM) of a LEC is an important element that makes a substantial contribution to the success of the LEC. In particular, the LEM enables energy sharing among prosumers, thereby offering a powerful and complete exploitation of the prosumer DERs within the community and reducing the grid dependence of the community. It also helps to increase the benefits for prosumers because the pricing proposed by the LEM is usually better than that of the electricity provider. As the conventional retail market is not suitable for the LEM, a new market has been studied in many previous studies to coordinate prosumers efficiently. Depending on the degree of decentralization, LEM structures can be classified into fully peer-to-peer (P2P) market and community-based (CB) market^16^. In P2P-LEM, there is no need for a central entity; prosumers are directly interconnected and trade bilaterally with one another. The main advantage of P2P-LEM is that it allows prosumers to have independent control of their trading^17^. The well-known pricing schemes that are generally used in this type of market are the alternating direction method of multipliers (ADMM)^18,19^ and game theory^20,21^. In the CB-LEM, there is a central entity that collects information of the trading and schedules of each prosumer and optimizes the overall profits in the market^22,23^. The interest of the community is predominant in this market, and certain prosumers may sacrifice their profits for the overall profit of the community. The mid-market rate (MMR)^24,25^ and auction-based^26,27^ pricing schemes are popular in this type of market.

### Related works

Numerous studies have been conducted on CEMSs. In this section, because our proposed method is day-ahead scheduling, we focus on reviewing existing day-ahead scheduling methods for a LEC.

In^10^, the authors proposed a residential community in which a load aggregator was used to collect all information of the DERs and loads at each home, and to construct an optimization problem to minimize the energy cost of the community. By solving this problem using the CPLEX solver, this load aggregator provided a day-ahead schedule for all DERs and appliances within the community. Every home of the community had its own RES and ESS. However, in this study, the authors assumed that solar power was only used for the community load, and it was not sold to the grid or stored in the ESS. Moreover, local energy trading was not supported in this community.

The authors of^28^ proposed day-ahead energy management for a community of 15 houses to minimize its energy cost. There were only three DERs that were shared among all members within the community: wind generation, solar photovoltaic (PV) generation, and an ESS. The RES energy could be used for the local load, stored in the ESS, or sold to the grid. The optimization problem of the community was solved by the particle swarm optimization (PSO) algorithm and a day-ahead schedule of sharing the DERs was provided. However, the individual DERs at each home were not considered.

In^23^, a community with an EMS, including a local P2P market and a user-dominated demand side response (UDDSR) program, was proposed to reduce the energy bills of the community. Certain homes within the community only had a PV system as the RES, and a central sharing ESS was integrated into the community to store surplus energy from its members. The UDDSR program collected flexible bids from the homes, including information of their controllable appliances. The program solved the MILP optimization problem and the schedules of controllable appliances were provided to maximize the load balancing between homes using the GUROBI solver. However, individual ESSs were not considered in this study.

In^14^, a day-ahead scheduling problem of a 24-home smart microgrid (SMG) was modeled as a multi-objective function to minimize the operation cost, the emission pollution, and the load curtailment cost. In this SMG, there was the SMG operator which controlled all communal DERs and different types of loads, consisting of shiftable load, curtailable load, and fixed load. Three types of communal DERs were considered, consisting of ESS, wind turbines, and diesel generator. A mixed integer nonlinear programming (MINLP) model for this scheduling problem was built and solved by using General Algebraic Modeling System (GAMS) software. However, the individual DERs at each home and local energy market between homes were not considered. Moreover, thermal satisfaction was not also considered.

In^12^, authors proposed a smart hybrid energy system (SHES) for energy management of a community. This system controlled communal DERs and different types of loads to minimize the operation cost and emission pollution, and to maximize the customer satisfaction. Five types of communal DERs were considered, consisting of PV systems, wind turbines, and diesel generator for electricity, and boiler and combined heat and power (CHP) for heating. A system model including all constraints and multi-objective function was built and solved by using shuffled frog leaping algorithm (SFLA). However, the individual DERs at each home and local energy market between homes were not considered.

In^13^, authors proposed a residential smart electrical distribution grid (RSEDG) in which an operator of RSEDG controlled all communal DERs and energy demands of customers (homes) inside a community. Four types of communal DERs were considered, consisting of PV systems, ESS, wind turbines, and diesel generator. A MINLP optimization model was built and solved by using GAMS software. Three objective functions were optimized, including minimization of the operation cost and emission pollution, minimization of the loss of load expectation, and minimization of the deviation between the demand curve and output power of RES. However, the individual DERs at each home and local energy market between homes were not considered. Moreover, thermal satisfaction was not also considered.

In^15^, day-ahead multi-objective optimal scheduling of a smart energy hub system (SEHS) for a community was proposed. The SEHS was an infrastructure comprised of all communal DERs and different types of customers (homes) of the community. Seven types of communal DERs were supported in this SEHS, consisting of PV systems, ESS, wind turbines, and diesel generator for electricity and boiler, CHP, and thermal storage system (TSS) for heating. Two types of customers were considered: responsive customers with shiftable load and non-responsive customer with fixed load. A MINLP optimization model for SEHS was built and solved by GAMS software. However, the individual DERs at each home and local energy market between homes were not considered.

In^3^, day-ahead scheduling of a prosumer-centric LEC using ADMM was proposed. In this study, each home had an individual PV system as the RES and an individual ESS. An optimization function to minimize the energy cost and power loss for each prosumer was built. Subsequently, the prosumers cooperated to solve the problem distributively by using ADMM algorithms. However, the RES energy was only used for home load and selling at each home; it could not be stored in the ESS. Moreover, this study only supported homes that included both an RES and ESS, and controllable load scheduling to satisfy user comfort was not considered.

In^2^, the authors proposed an energy sharing framework for a community in which each building had its own DERs, including an RES and ESS. Each building first optimized its DERs and controllable load to minimize the energy cost without energy sharing. Thereafter, a non-cooperative sharing game was used to determine the energy sharing profile and corresponding payments of each building. However, in this study, the RES energy could not be stored in the ESS, and homes that had an RES only or ESS only were not considered.

The authors of^29^ proposed a two-stage EMS for a group of buildings. In the day-ahead stage, an MILP-based objective function of the scheduling model was constructed to minimize the energy cost while maintaining the user comfort for each building. Subsequently, in the real-time stage, each building participated in a transactive market to maximize the profits. However, in this study, every building was equipped with solar PV and batteries. Moreover, no energy was sold to the public grid or traded with other homes in the day-ahead stage.

Motivated by the above works, we propose a community with a system-centric CEMS that supports different types of homes with individual DERs: homes with both an RES and ESS, homes with an individual RES only or individual ESS only, and homes without an RES or ESS. Moreover, individual RES energy can be used in many manners: for home loads, for selling to outside the community, or for charging to the individual ESS of a home if this home has its own ESS. In our community, local energy trading between prosumers is also supported using the MMR pricing scheme. The proposed model of this paper is compared with other models listed in the related works in Table 1.

**Table 1:** A comparison between this paper and the papers mentioned in the related works.

Work	Type of CEMS	Algorithm (technique)	RES		ESS		Local energy market	Supporting different types of homes	Thermal satisfaction
			Communal	Individual	Communal	Individual			
^10^	System-centric	MILP		$\checkmark$		$\checkmark$			$\checkmark$
^28^	System-centric	PSO	$\checkmark$		$\checkmark$				
^23^	System-centric	MILP and P2P market		$\checkmark$	$\checkmark$		$\checkmark$		
^14^	System-centric	MINLP			$\checkmark$				
^12^	System-centric	SFLA	$\checkmark$						$\checkmark$
^13^	System-centric	MINLP	$\checkmark$		$\checkmark$				
^15^	System-centric	MINLP	$\checkmark$		$\checkmark$				$\checkmark$
^3^	Prosumer-centric	MILP and ADMM		$\checkmark$		$\checkmark$	$\checkmark$		
^2^	Prosumer-centric	MILP and game theory		$\checkmark$		$\checkmark$	$\checkmark$		
^29^	Prosumer-centric	MILP and Transactive Market		$\checkmark$		$\checkmark$	$\checkmark$		$\checkmark$
Our work	System-centric	MILP and MMR		$\checkmark$		$\checkmark$	$\checkmark$	$\checkmark$	$\checkmark$

### Contribution

We focus on investigating an optimization problem for a system-centric CEMS to minimize the overall energy cost of a LEC that supports different types of homes and the CB-LEM between them. In our LEC, four types of smart homes are supported: homes with an RES and ESS, homes with an RES only, homes with an ESS only, and normal homes without an RES or ESS. A CB-LEM between smart homes is also built to exploit the individual RESs and ESSs fully. The RES energy of a home is used in many manners: for home devices, for storage in the ESS of the home, or to sell to the community. Likewise, the ESS energy of a home is used for the home load or for selling to the community. The ESS is also used to store RES energy or cheap energy from the community. Based on the functionalities of the RES and ESS, general mathematical formulas of the DERs and load for each home and the entire community are constructed and successfully transformed into an MILP problem that can be solved in a short time. The performance of our proposed CEMS is evaluated by applying to different communities with different number of homes. A community with a prosumer-centric CEMS is also modeled and compared with our proposed CEMS. Moreover, we also demonstrate the trade-off of the MMR pricing in the local energy trading of the community. This trade-off allows the benefits of different types of homes to be flexibly changed. In conclusion, the main contributions of this study can be listed as follows:

- We propose a novel system-centric CEMS for a community that includes four different types of homes and supports a CB-LEM to exploit the RESs and ESSs of the homes within the community fully.
- We construct general mathematical formulas of the DERs and load for each home and for the entire community. Based on these formulas, a day-ahead optimization problem of the community is built and successfully transformed into an MILP problem that can be solved in a short time. The optimization of the daily energy cost of the community and satisfaction of thermal comfort are achieved by solving this problem. Compared to the community with a prosumer-centric CEMS, the community with our proposed CEMS only has 78% of the daily energy cost.
- Our proposed CEMS is analyzed extensively with a variety of case studies and we also analyze the trade-off of the MMR pricing in the local energy trading of the community in detail.

### Paper organization

The remainder of this paper is organized as follows: In “System model and problem formulation” section, the construction and transformation of our system model and the optimization problem are presented in detail. “Local energy trading mechanism” section describes the local energy trading mechanism with MMR pricing. “Prosumer-centric CEMS for community” section builds the mathematical model of the prosumer-centric CEMS for a community. In “Simulations and results” section, the simulations and results are discussed. Some ideas are discussed in “Discussion” section. Finally, “Conclusions and future works” section outlines the conclusions and potential future works.

## System model and problem formulation

**Figure 1:**
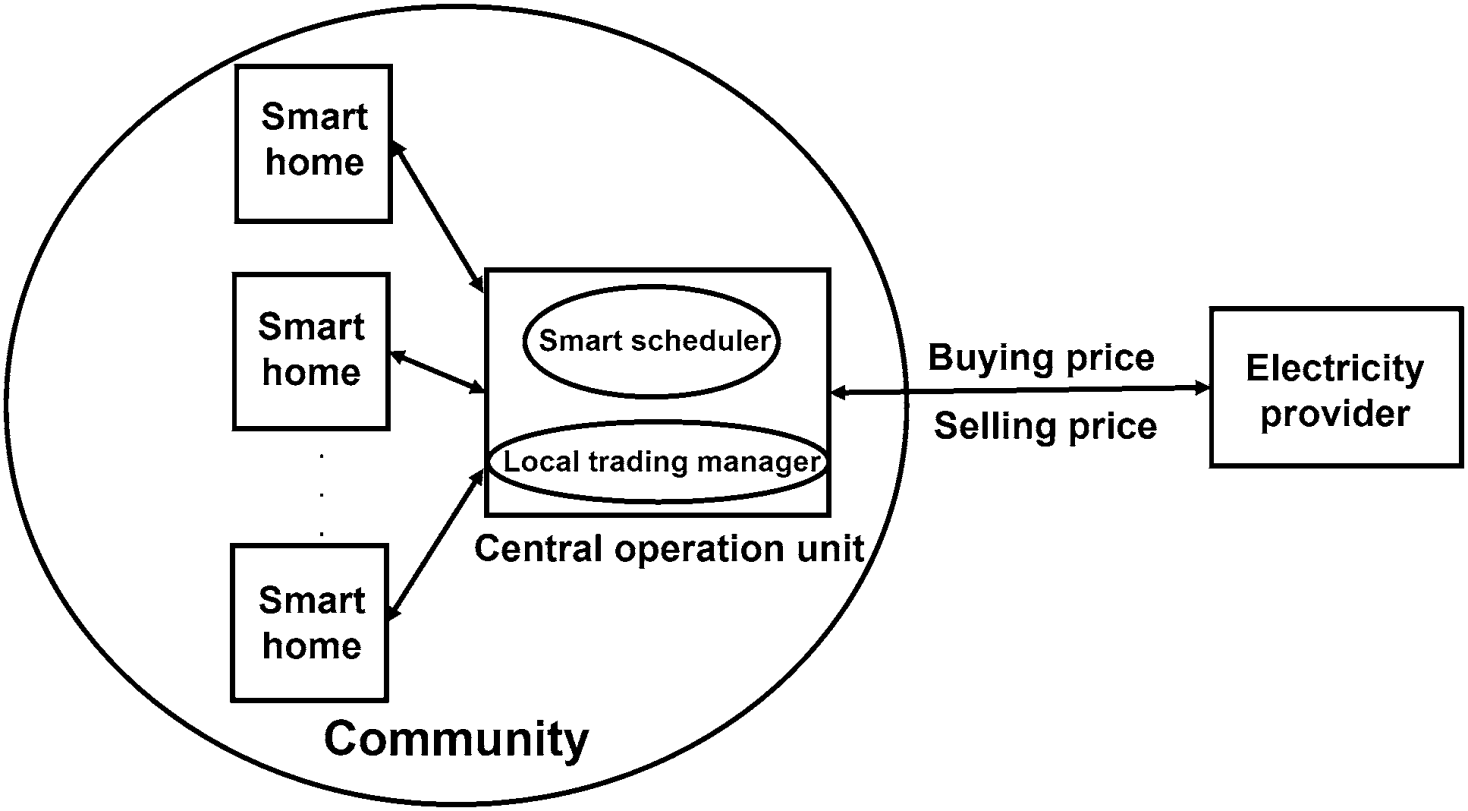
Structure of community energy management.

In this section, we consider a community including a group of smart homes $H=\{1,2,\ldots,N\}$, as illustrated in Fig. 1. Within the community, the central operation unit collects information from all homes and from the electricity provider (EP), such as the DERs of each home, price information, forecast temperature, and solar irradiation. An smart scheduler (SS) that includes an optimization algorithm and local trading manager that is responsible for coordinating the local trading within the community are installed inside the central operation unit. At the beginning of the day, useful information is received from the homes and EP. Subsequently, the SS is run to create an optimized schedule for all DERs and devices of each home during a day, whereas the local energy trading manager is run to calculate local buying and selling prices. From these local prices, the daily energy cost of each home inside the community is determined.

**Figure 2:**
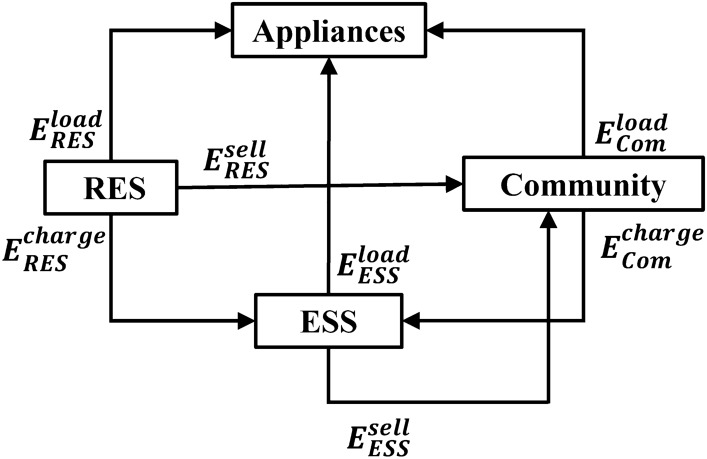
Energy flows in a smart home with RES and ESS.

Each home may have both ESS and RES, only one of these, or neither. The energy flows of a home, which includes both the ESS and RES, in the community are depicted in Fig. 2. If a home has an RES, its energy can be used for home loads, ESS charging, or selling to the community. If a home has an ESS, its energy is used for home loads and selling to the community. The ESS is also used to store electricity from the community at a low price and to provide electricity for home loads at the high price time. We consider two different loads in a home for each time slot: the fixed load and controllable load. Fixed loads are the loads of devices for which the power consumption cannot be changed in this time slot, whereas controllable loads are the loads of devices for which the power consumption can be changed to satisfy certain constraints based on the environmental status.

We build mathematical formulas and constraints for all RESs, ESSs, and loads in the community during a day from 0 AM to 12 PM to optimize the daily energy cost of the community. We also divide a day into $T=24$ time slots, and the duration of each time slot is $\Delta t = 1$ h.

### Controllable load

In this study, an adjusted HVAC system in heating mode is considered as the controllable load in a home. Let $p_{i}(t)$ be the input power of the HVAC system in time slot *t* at home *i*. This variable can be adjusted continuously within a certain range^30^.

1 $$\begin{aligned} 0 \le p_{i}(t) \le P_{max}, \end{aligned}$$

where $P_{max}$ is the rating power of the HVAC system, and the HVAC energy $E_{i,HVAC}(t) \ge 0$ that is required in time slot *t* at home *i* can be calculated as follows:

2 $$\begin{aligned} E_{i,HVAC}(t) = p_{i}(t) \cdot \Delta t. \end{aligned}$$

According to^30,31^, and^32^, the indoor temperature $T_{i}^{in}(t+1)$ of home *i*, which is influenced by the input power of the HVAC system $p_{i}(t)$, the indoor temperature $T_{i}^{in}(t)$, and the outdoor temperature $T^{out}(t)$, is calculated as follows:

3 $$\begin{aligned} T_{i}^{in}(t+1) = \varepsilon \cdot T_{i}^{in}(t) + (1- \varepsilon ) \cdot (T^{out}(t) + \frac{\eta \cdot p_{i}(t)}{A}), \end{aligned}$$

where $\varepsilon$ is a constant that describes the system inertia. Furthermore, $\eta$ refers to the thermal conversion efficiency and *A* is the overall thermal conductivity.

When using an HVAC system, the indoor temperature in each time slot of a day must be guaranteed in a comfortable temperature range $[T_{min}, T_{max}]$ at each home in the community. Hence, we obtain the following constraint:

4 $$\begin{aligned} T_{min} \le T_{i}^{in}(t) \le T_{max}. \end{aligned}$$

### ESS model

Let $E_{i,ESS}^{Level}(t)$ be the energy level of the ESS of home *i* after time slot *t*. As described in Fig. 2, with $\forall t, 1 \le t \le T$, we obtain the following formula:

5 $$\begin{aligned} E_{i,ESS}^{Level}(t) &=E_{i,ESS}^{Level}(t-1) \nonumber \\ & \quad - \big (E_{i,ESS}^{load}(t) + E_{i,ESS}^{sell}(t) \big ) / \eta ^{ESS} \nonumber \\ & \quad + \big (E_{i,RES}^{charge}(t) + E_{i,Com}^{charge}(t)\big ) \cdot \eta ^{ESS} \end{aligned}$$

where $E_{i,ESS}^{load}(t) \ge 0$ is the energy from the ESS of home *i* that is used for appliances in time slot *t*. $E_{i,ESS}^{sell}(t) \ge 0$ is the energy from the ESS of home *i* that is used for selling back to the community in time slot *t*. $E_{i,RES}^{charge}(t) \ge 0$ is the energy that is stored in the ESS from the RES of home *i* in time slot *t*. $E_{i,Com}^{charge}(t) \ge 0$ is the energy that is stored in the ESS of home *i* from the community in time slot *t*. $\eta ^{ESS}$ is the ESS efficiency.

When using the ESS, the following constraints must be satisfied:

6 $$\begin{aligned}&EL_{i,min} \le E_{i,ESS}^{Level}(t) \le EL_{i,max} \end{aligned}$$

7 $$\begin{aligned}&E_{i,RES}^{charge}(t) + E_{i,Com}^{charge}(t) \le Ch_{i,rate} \cdot \Delta t \cdot mode_{i,ESS}(t) \end{aligned}$$

8 $$\begin{aligned}&E_{i,ESS}^{load}(t) + E_{i,ESS}^{sell}(t) \le Dh_{i,rate} \cdot \Delta t \cdot \big (1 - mode_{i,ESS}(t)\big ) \end{aligned}$$

9 $$\begin{aligned}&mode_{i,ESS}(t) = {\left\{ \begin{array}{ll} 1 &{} \quad \text {if ESS is charged in slot } t \\ 0 &{} \quad \text {if ESS is discharged in slot } t \end{array}\right. } \end{aligned}$$

where $EL_{i,min}$ and $EL_{i,max}$ are the minimum and maximum energy levels of the ESS of home *i*, respectively. $Ch_{i,rate}$ and $Dh_{i,rate}$ are the maximum charge and discharge rates of the ESS, respectively. $mode_{i,ESS}(t)$ is a binary variable to avoid simultaneous ESS charging and discharging in time slot *t* at home *i*. It is assumed that the ESS cannot be charged and discharged simultaneously.

As we only consider our system during a day (with no net accumulation for the following day), the energy level should be returned to the initial energy level $EL_{i,0}$ at the end of the day. Thus, we have

10 $$\begin{aligned} E_{i,ESS}^{Level}(T) = EL_{i,0}. \end{aligned}$$

### RES model

According to^33^ and^34^, the output energy $E_{i,RES}(t) \ge 0$ from a PV system of home *i* in time slot *t* ($1 \le t \le T$) in kWh can be measured as

11 $$\begin{aligned} E_{i,RES}(t)= GHI_i(t) \cdot S_i \cdot \eta ^{RES} \cdot \Delta t, \end{aligned}$$

where $S_i$ is the total area $(\text{m}^2)$ of the solar panels of home *i*. $GHI_i(t)$ is the global horizontal irradiation $(\text{kW}/\text{m}^2)$ at the location of the solar panels of home *i* in time slot *t*. $\eta ^{RES}$ is the solar conversion efficiency of the PV system.

As illustrated in Fig. 2, this energy can be used for appliances, ESS charging, and selling to the community. Thus, we determine the following constraint:

12 $$\begin{aligned} E_{i,RES}^{load}(t) + E_{i,RES}^{charge}(t) + E_{i,RES}^{sell}(t) = E_{i,RES}(t), \end{aligned}$$

where $E_{i,RES}^{load}(t) \ge 0$ is the energy from the RES of home *i* that is used for the appliances of this home in time slot *t*, and $E_{i,RES}^{sell}(t) \ge 0$ is the energy from the RES of home *i* that is sold back to the community in time slot *t*.

### Energy balancing

To maintain the energy balance in a smart home, the total energy demand should be equal to the total energy supply. Hence, as shown in Fig. 2, with $\forall t, 1 \le t \le T$, we obtain

13 $$\begin{aligned} E_{i,Fix}(t) + E_{i,HVAC}(t) =&E_{i,Com}^{load}(t) + E_{i,ESS}^{load}(t) \nonumber \\&+ E_{i,RES}^{load}(t), \end{aligned}$$

where $E_{i,Fix}(t) \ge 0$ is the fixed load of home *i* in time slot *t*. Moreover, $E_{i,Com}^{load}(t) \ge 0$ is the energy that home *i* requires for the home load from the community in time slot *t*.

### Avoiding simultaneous buying and selling of home

In time slot *t*, let $E_{i,Com}^{buy}(t) \ge 0$ and $E_{i,Com}^{sell}(t) \ge 0$ be the total buying energy and selling energy of a home from/to the community, respectively. Thus, we obtain

14 $$\begin{aligned}&E_{i,Com}^{buy}(t) = E_{i,Com}^{load}(t) + E_{i,Com}^{charge}(t). \end{aligned}$$

15 $$\begin{aligned}&E_{i,Com}^{sell}(t) = E_{i,RES}^{sell}(t) + E_{i,ESS}^{sell}(t). \end{aligned}$$

Residents are unable to buy and sell from/to the community simultaneously within a time slot. Hence, $E_{i,Com}^{buy}(t)$ in (14) and $E_{i,Com}^{sell}(t)$ in (15) have the following constraints:

16 $$\begin{aligned}&0 \le E_{i,Com}^{buy}(t) \le M \cdot mode_{i,home}(t) \end{aligned}$$

17 $$\begin{aligned}&0 \le E_{i,Com}^{sell}(t) \le M \cdot \big (1 - mode_{i,home}(t)\big ) \end{aligned}$$

18 $$\begin{aligned}&mode_{i,home}(t) = {\left\{ \begin{array}{ll} 1 &{} \text {if home i buys energy from community } \\ 0 &{} \text {if home i sells energy to community }, \\ \end{array}\right. } \end{aligned}$$

where *M* is a very large value that $E_{i,Com}^{buy}(t)$ and $E_{i,Com}^{sell}(t)$ never exceed; e.g., $M=10^9$. $mode_{i,home}(t)$ is a binary variable and its value is depended on the buying/selling status of home *i*.

Because $mode_{i,home}(t)$ is a binary variable, constraints (16) and (17) guarantee that in the time slot *t*, both variable $E_{i,Com}^{buy}(t)$ and $E_{i,Com}^{sell}(t)$ cannot be larger than 0 simultaneously. Only one of them is larger than 0 and another variable must be equal to 0. In other words, (16), (17), and (18) help to avoid the simultaneous buying and selling of home *i*.

### Optimization problem of community

In time slot *t*, net energy of home *i*, $E_{i,Com}(t)$ which residents need to buy/sell from/to the community, is

19 $$\begin{aligned} E_{i,Com}(t) = E_{i,Com}^{buy}(t) - E_{i,Com}^{sell}(t). \end{aligned}$$

In time slot *t*, from (19), the total net energy of the community with *N* homes, which it need to buy/sell from/to the EP, is

20 $$\begin{aligned} E_{Com}(t) = \displaystyle \sum _{i=1}^{N}E_{i,Com}(t) = \displaystyle \sum _{i=1}^{N}E_{i,Com}^{buy}(t) - \displaystyle \sum _{i=1}^{N}E_{i,Com}^{sell}(t). \end{aligned}$$

If $E_{Com}(t) > 0$, the total buying energy is larger than the total selling energy within the community and it needs to buy an energy quantity $E_{Com}(t)$ from the EP. If $E_{Com}(t) < 0$, the total buying energy is smaller than the total selling energy within the community and it needs to sell an energy quantity $|E_{Com}(t)|$ to the EP. If $E_{Com}(t) = 0$, the total buying energy is equal to the total selling energy within the community.

Let $P_{MG}(t)$ be the day-ahead buying price at which the community buys electricity from the EP in time slot *t*. Because day-ahead selling price at which the community sells electricity to the EP is smaller than day-ahead buying price $P_{MG}(t)$. Hence, in time slot *t*, the day-ahead selling price can be determined as $\alpha \cdot P_{MG}(t)$ with $0< \alpha < 1$. Let $C_{Com}(t)$ be the energy cost of the community in time slot *t*. From (20), the optimization problem of the community that minimizes the daily energy cost of the community can be formulated as

21 $$\begin{aligned}&min \displaystyle \sum _{t=1}^{T} C_{Com}(t)= min \displaystyle \sum _{t=1}^{T} P(t) \cdot E_{Com}(t) \nonumber \\&\quad = min \displaystyle \sum _{t=1}^{T}P(t) \cdot \Big (\displaystyle \sum _{i=1}^{N}E_{i,Com}^{buy}(t) - \displaystyle \sum _{i=1}^{N}E_{i,Com}^{sell}(t)\Big ), \end{aligned}$$

where

22 $$\begin{aligned} P(t) = {\left\{ \begin{array}{ll} P_{MG}(t) &{} \text {if } \displaystyle \sum _{i=1}^{N}E_{i,Com}^{buy}(t) > \displaystyle \sum _{i=1}^{N}E_{i,Com}^{sell}(t) \\ \alpha \cdot P_{MG}(t) &{} \text {if } \displaystyle \sum _{i=1}^{N}E_{i,Com}^{buy}(t) < \displaystyle \sum _{i=1}^{N}E_{i,Com}^{sell}(t) \end{array}\right. } \end{aligned}$$

subject to:

23 $$\begin{aligned}&(1){-}(18) \end{aligned}$$

24 $$\begin{aligned}&\quad -E_{max} \le \displaystyle \sum _{i=1}^{N}E_{i,Com}^{buy}(t) - \displaystyle \sum _{i=1}^{N}E_{i,Com}^{sell}(t) \le E_{max}, \end{aligned}$$

where formula (22) shows that if the community buys energy from EP, the price of buying is equal to $P_{MG}(t)$, and if the community sells energy to EP, the price of selling is only equal to price $\alpha \cdot P_{MG}(t)$. Constraint (24) is added to restrict the total net energy of the community to be lower than an energy peak $E_{max}$ (the capacity of the power cable).

### Linear transformation

Owing to the definition of variable *P*(*t*) in (22), our optimization problem in (21) is a nonlinear function that can be solved using numerous well-known nonlinear solvers and heuristic algorithms. However, these usually require substantial computational time, especially for a large community including many homes. Hence, in this section, we describe a means of transforming our optimization problem into an MILP problem.

To remove variable *P*(*t*), we introduce a new variable *s*(*t*), which is a binary variable that indicates the buying/selling status of the community from/to the EP at each time slot *t*.

25 $$\begin{aligned} s(t) = {\left\{ \begin{array}{ll} 1 &{} \quad \text {if } \displaystyle \sum _{i=1}^{N}E_{i,Com}^{buy}(t) > \displaystyle \sum _{i=1}^{N}E_{i,Com}^{sell}(t) \\ 0 &{} \quad \text {if } \displaystyle \sum _{i=1}^{N}E_{i,Com}^{buy}(t) < \displaystyle \sum _{i=1}^{N}E_{i,Com}^{sell}(t) \end{array}\right. } \end{aligned}$$

The meaning of formula (25) is that if the community buys energy from EP, the value of variable *s*(*t*) should be 1 (buying status) and if the community sells energy to EP, the value of variable *s*(*t*) should be 0 (selling status). To accomplish this setting, we determine the following constraints for variable *s*(*t*):

26 $$\begin{aligned}&\displaystyle \sum _{i=1}^{N}E_{i,Com}^{buy}(t) \ge \displaystyle \sum _{i=1}^{N}E_{i,Com}^{sell}(t) - M \cdot (1-s(t)) \end{aligned}$$

27 $$\begin{aligned}&\displaystyle \sum _{i=1}^{N}E_{i,Com}^{buy}(t) \le \displaystyle \sum _{i=1}^{N}E_{i,Com}^{sell}(t) + M \cdot s(t), \end{aligned}$$

where *M* is a very large value that $\displaystyle \sum \nolimits _{i=1}^{N}E_{i,Com}^{buy}(t)$ and $\displaystyle \sum \nolimits _{i=1}^{N}E_{i,Com}^{sell}(t)$ never exceed; e.g., $M=10^9$.

By using the variable *s*(*t*), our optimization problem can be transformed into

28 $$\begin{aligned} min \displaystyle \sum _{t=1}^{T} C_{Com}(t), \end{aligned}$$

subject to:

29 $$\begin{aligned}&C_{Com}(t) \ge \left(\displaystyle \sum _{i=1}^{N}E_{i,Com}^{buy}(t) - \displaystyle \sum _{i=1}^{N}E_{i,Com}^{sell}(t)\right) \cdot P_{MG}(t) - M \cdot (1-s(t)) \end{aligned}$$

30 $$\begin{aligned}&C_{Com}(t) \le \left (\displaystyle \sum _{i=1}^{N}E_{i,Com}^{buy}(t) - \displaystyle \sum _{i=1}^{N}E_{i,Com}^{sell}(t)\right) \cdot P_{MG}(t) + M \cdot (1-s(t)) \end{aligned}$$

31 $$\begin{aligned}&C_{Com}(t) \ge \left(\displaystyle \sum _{i=1}^{N}E_{i,Com}^{buy}(t) - \displaystyle \sum _{i=1}^{N}E_{i,Com}^{sell}(t)\right) \cdot \alpha \cdot P_{MG}(t) - M \cdot s(t) \end{aligned}$$

32 $$\begin{aligned}&C_{Com}(t) \le \left(\displaystyle \sum _{i=1}^{N}E_{i,Com}^{buy}(t) - \displaystyle \sum _{i=1}^{N}E_{i,Com}^{sell}(t)\right) \cdot \alpha \cdot P_{MG}(t) + M \cdot s(t) \end{aligned}$$

and

33 $$\begin{aligned} (1){-}(18), (24), (26), (27), (29){-}(32). \end{aligned}$$

The meanings of above constraints is that if the community buys energy from EP ($s(t)=1$), the constraints (29) and (30) will set $C_{Com}(t)$ to a value $\left(\displaystyle \sum \nolimits_{i=1}^{N}E_{i,Com}^{buy}(t) - \displaystyle \sum \nolimits _{i=1}^{N}E_{i,Com}^{sell}(t)\right) \cdot P_{MG}(t)$. If the community sells energy to EP ($s(t)=0$), the constraints (31) and (32) will set $C_{Com}(t)$ to a value $\left(\displaystyle \sum \nolimits_{i=1}^{N}E_{i,Com}^{buy}(t) - \displaystyle \sum \nolimits _{i=1}^{N}E_{i,Com}^{sell}(t)\right) \cdot \alpha \cdot P_{MG}(t)$.

It is clear that problem (28) is an MILP problem that can be solved by many advanced mathematical solvers in a short time. The output of solving the problem is the day-ahead schedule of all DERs and HVACs in the community to minimize the daily energy cost of the community and satisfy the thermal comfort in every home.

## Local energy trading mechanism

In this section, we introduce local energy trading among the homes of the community. This trading function is run after the SS is run, and each home already knows the buying or selling action at each time slot of the day. As illustrated in Fig. 1, the residents of the homes first trade energy with others in the community using the local buying and selling prices, instead of trading directly with the EP. After trading together within the community, if the community requires more energy or has surplus energy to sell, the community trades directly with the EP. To encourage energy trading among the homes of the community, the local buying/selling prices that are proposed by the local trading manager of the central operation unit should be smaller/larger than the buying/selling prices that are proposed by the EP. The pricing in this study is the MMR pricing, which is adopted from^1,24^.

### MMR pricing

Let $P_{LB}(t), P_{LS}(t)$ be the local buying and selling prices at time slot *t*, respectively. We must have $P_{LB}(t) \le P_{MG}(t)$ and $P_{LS}(t) \ge \alpha \cdot P_{MG}(t)$. Let $P_{mid}(t)$ be a price that has a value in the range from the selling price to buying price proposed by the EP.

34 $$\begin{aligned} \alpha \cdot P_{MG}(t) \le P_{mid}(t) \le P_{MG}(t) \end{aligned}$$

In this study, we use $P_{mid}(t)= (1+\alpha )\cdot P_{MG}(t)/2$. At each time slot *t*, the local buying and selling prices depend on the variable $E_{Com}(t)$ of the community, and they can be calculated as follows.

1. $E_{Com}(t)=0$: Within the community, the total buying energy is equal to the total selling energy. The local buying and selling prices at this time slot are equal to the average price $P_{mid}(t)$.
  35 $$\begin{aligned} P_{LB}(t) = P_{LS}(t) = P_{mid}(t) \end{aligned}$$
2. $E_{Com}(t) > 0$: Within the community, the total buying energy is larger than the total selling energy. The community needs to buy an energy quantity $E_{Com}(t)$ from the EP at buying price $P_{MG}(t)$. In this case, the local selling and buying prices in this time slot will be
  36 $$\begin{aligned}&P_{LS}(t) = P_{mid}(t) \end{aligned}$$
  37 $$\begin{aligned}&P_{LB}(t) = \frac{P_{LS}(t) \displaystyle \sum \nolimits_{i=1}^{N}E_{i,Com}^{sell}(t) + P_{MG}(t) E_{Com}(t)}{\displaystyle \sum \nolimits_{i=1}^{N}E_{i,Com}^{buy}(t)}. \end{aligned}$$
  where $P_{LS}(t) \displaystyle \sum\nolimits _{i=1}^{N}E_{i,Com}^{sell}(t)$ is the total energy cost which all buying homes must pay for all selling homes. $P_{MG}(t) E_{Com}(t)$ is the energy cost which all buying homes must pay for EP. $\displaystyle \sum \nolimits_{i=1}^{N}E_{i,Com}^{buy}(t)$ is the total energy quantity which all buying homes must buy. The value of $P_{LB}(t)$, calculated in (37), is based on the fact that the total buying energy cost is needed to proportionally share among all buying homes according to their buying energy quantity.
3. $E_{Com}(t) < 0$: Within the community, the total buying energy is smaller than the total selling energy. The community needs to sell an energy quantity $|E_{Com}(t)|$ to the EP at selling price $\alpha \cdot P_{MG}(t)$. In this case, the local buying and selling prices in this time slot will be
  38 $$\begin{aligned}&P_{LB}(t) = P_{mid}(t) \end{aligned}$$
  39 $$\begin{aligned}&P_{LS}(t) = \frac{P_{LB}(t) \displaystyle \sum \nolimits_{i=1}^{N}E_{i,Com}^{buy}(t) + \alpha P_{MG}(t) |E_{Com}(t)|}{\displaystyle \sum \nolimits_{i=1}^{N}E_{i,Com}^{sell}(t)}. \end{aligned}$$
  where $P_{LB}(t) \displaystyle \sum \nolimits_{i=1}^{N}E_{i,Com}^{buy}(t)$ is the selling profit (or energy cost) which all selling homes receive from all buying homes. $\alpha P_{MG}(t) |E_{Com}(t)|$ is the selling profit which all selling homes receive from EP. $\displaystyle \sum \nolimits _{i=1}^{N}E_{i,Com}^{sell}(t)$ is the total selling energy quantity which all selling homes sold. The value of $P_{LS}(t)$, calculated in (39), is based on the fact that the total selling profit is needed to proportionally share among the selling homes according to their selling energy quantity.

With this pricing scheme, it is clear that the local buying and selling prices within our community are always better than the buying and selling prices that are proposed by the EP. Hence, to improve the benefit for each home, the central operation unit prefers the trading of energy among its homes over trading with the EP. Given the local buying and selling prices, the daily energy cost of home *i* can be calculated as follows:

40 $$\begin{aligned} C_{i}= \displaystyle \sum _{t=1}^{T} P_{L}(t) \cdot E_{i,Com}(t), \end{aligned}$$

where

41 $$\begin{aligned} P_{L}(t) = {\left\{ \begin{array}{ll} P_{LB}(t) &{} \quad \text {if } E_{i,Com}(t) > 0 \\ P_{LS}(t) &{} \quad \text {if } E_{i,Com}(t) < 0. \end{array}\right. } \end{aligned}$$

## Prosumer-centric CEMS for community

We describe a prosumer-centric CEMS for a community for comparison with our proposed CEMS in this section. As discussed in “Introduction” section, in a prosumer-centric CEMS, each prosumer is first allowed to optimize their own optimization problem without energy trading. After solving this problem, a day-ahead schedule of their individual DERs is provided, following which they join a local energy market to receive further benefits.

In the first stage, the optimization problem of each home in the community is as follows:

42 $$\begin{aligned}&min \displaystyle \sum _{t=1}^{T} P_{i}(t) \cdot E_{i,Com}(t) \nonumber \\&\quad = min \displaystyle \sum _{t=1}^{T}P_{i}(t) \cdot \Big (E_{i,Com}^{buy}(t) - E_{i,Com}^{sell}(t)\Big ), \end{aligned}$$

where

43 $$\begin{aligned} P_{i}(t) = {\left\{ \begin{array}{ll} P_{MG}(t) &{} \quad \text {if } E_{i,Com}(t) >0 \\ \alpha \cdot P_{MG}(t) &{} \quad \text {if } E_{i,Com}(t) < 0 \end{array}\right. } \end{aligned}$$

subject to:

44 $$\begin{aligned}&(1) {-} (18) \end{aligned}$$

45 $$\begin{aligned}&-E_{i,max} \le E_{i,Com}^{buy}(t) - E_{i,Com}^{sell}(t) \le E_{i,max}, \end{aligned}$$

where formula (43) shows that if home *i* buys energy from EP, the price of buying is equal to $P_{MG}(t)$, and if home *i* sells energy to EP, the price of selling is only equal to $\alpha \cdot P_{MG}(t)$. Constraint (45) is added to restrict the net energy of the home *i* to be lower than the energy peak $E_{i,max}$.

As a home cannot simultaneously buy and sell energy in a time slot (constraints (16) and (17)), two variables $E_{i,Com}^{buy}(t)$ and $E_{i,Com}^{sell}(t)$ cannot be larger than 0 simultaneously. Only one of them is larger than 0 and another variable must be 0 in the time slot *t*. Hence, the problem in (42) can be converted into

46 $$\begin{aligned} min \displaystyle \sum _{t=1}^{T}\Big (E_{i,Com}^{buy}(t) \cdot P_{MG}(t) - E_{i,Com}^{sell}(t) \cdot \alpha \cdot P_{MG}(t) \Big ) \end{aligned}$$

subject to:

47 $$\begin{aligned} (1){-}(18), (45). \end{aligned}$$

It is clear that the problem in (46) is an MILP problem that can be solved by many solvers. The output is the day-ahead schedule of the DERs and HVAC of the home.

In the second stage, based on this day-ahead schedule, each prosumer joins a local energy market. For comparison, the local energy market with the MMR pricing described in “Local energy trading mechanism” section is reused in this CEMS.

## Simulations and results

In this section, a community consisting of a group of 10 homes is simulated under the control of different CEMSs: the proposed CEMS, a prosumer-centric CEMS, and no CEMS in which each home trades directly with the EP, to verify the efficiency and the performance of our proposed CEMS.

**Table 2:** PV system and ESS at each home.

Device	Home 1	Home 2	Home 3	Home 4	Home 5	Home 6	Home 7	Home 8	Home 9	Home 10
PV ($\text{m}^2$)	10	10	10	5	5	X	X	X	X	X
ESS (kWh)	10	10	10	X	X	5	5	X	X	X

### Simulation setup

**Table 3:** Main parameters of HVAC system and environment.

Parameter	Value
$T_{min}$	66.2 °F (19 °C)
$T_{max}$	75.2 °F (°C)
$P_{max}$	15 kW
$\varepsilon$	0.7^32^
$\eta$	2.5^32^
*A*	0.14 kW/°F^32^

**Figure 3:**
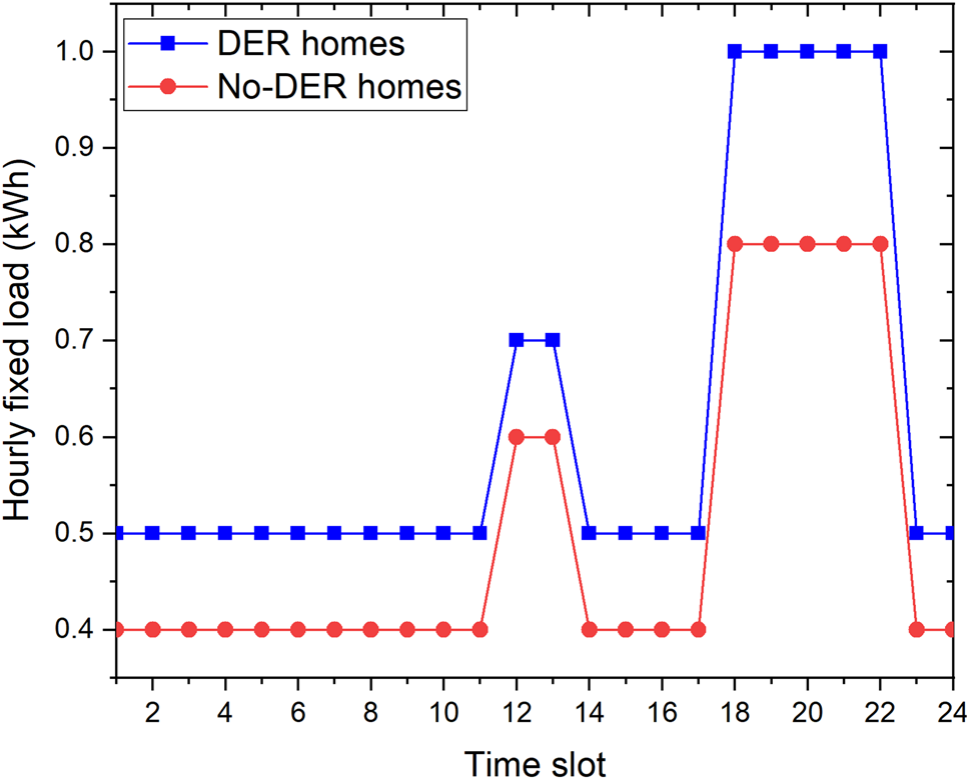
Hourly fixed loads of DER homes and no-DER homes.

Our proposed community consists of 10 homes in Detroit city, Michigan, USA. Each home has its own HVAC as the controllable load and the parameters of the HVAC are the same for every home, as indicated in Table 3. For the ESS and RES, we assume that three homes have an ESS and a PV system: *Home 1, Home 2, and Home 3*; two homes only have a PV system: *Home 4 and Home 5*; two homes only have an ESS: *Home 6 and Home 7*; and three homes have neither: *Home 8, Home 9 and Home 10*, as listed in Tables 2, 3. The numbers in this Table indicate the area of the solar panels $S_i$ and the maximum level of the ESS $EL_{i,max}$ at each home. The initial level $EL_{i,0}$ and minimum level $EL_{i,min}$ of the ESS at each home are the same, and $EL_{i,min} = EL_{i,0}=0.5$ kWh. The maximum charging rate $Ch_{i,rate}$ and maximum discharging rate $Dh_{i,rate}$ of the ESS at each home are the same, and $Ch_{i,rate}=Dh_{i,rate}=2$ kW. $\eta ^{ESS} = \eta ^{RES}=0.9$. The hourly fixed loads of the DER homes that have at least one DER (*Home 1 to Home 7*) and the homes without DERs (*Home 8 to Home 10*) used in our simulation are shown in Fig. 3. It is worth noting that our simulations can be run well with any values of hourly fixed loads of homes in the real life.

**Figure 4:**
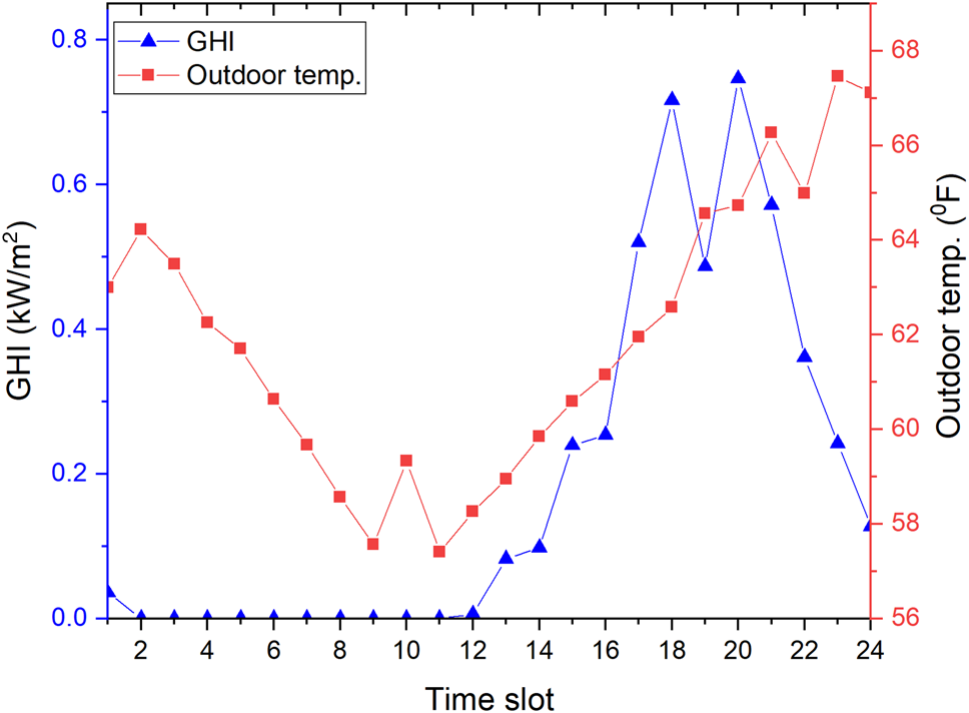
Day-ahead GHI and outside temperature of day in Detroit city.

**Figure 5:**
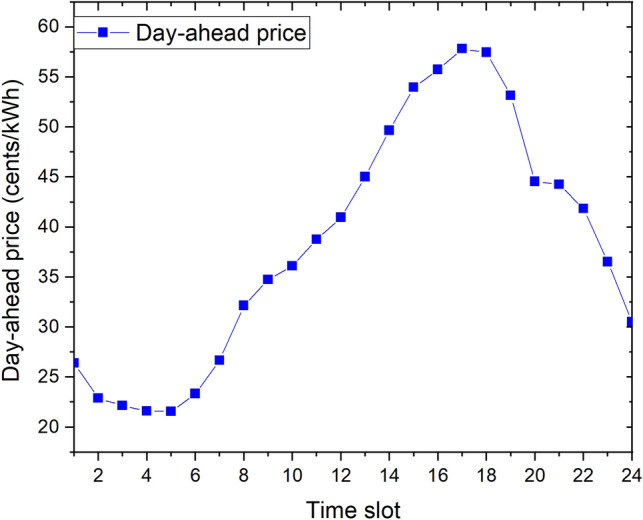
Day-ahead prices of day in Michigan state.

The day-ahead GHI from a photovoltaic geographical information system database^35^ and day-ahead outside temperature from the Kaggle website^36^ of a day in Detroit city, Michigan state, USA are presented in Fig. 4. The day-ahead hourly prices $P_{MG}(t)$ of the EP in Detroit city, which were extracted from the Pecan Street database^37^, are also indicated in Fig. 5. We assume that the day-ahead selling price from the community to the EP is equal to $80\%$ of this price at every time slot ($\alpha = 0.8$).

All of our simulations for the three scenarios were built and run using the mathematical programming software AIMMS *v*4.82^38^ with CPLEX *v*20.1 installed on an Intel(R) Core(TM) i7-8700 CPU @ 3.20GHz and 16 GB RAM with Windows 10 Pro (64-bit). AIMMS is a high-level modeling commercial software that integrates advanced mathematical solvers such as CPLEX and Conopt for solving LP, MILP, and MINLP problems.

### Energy cost of community with three CEMSs

**Figure 6:**
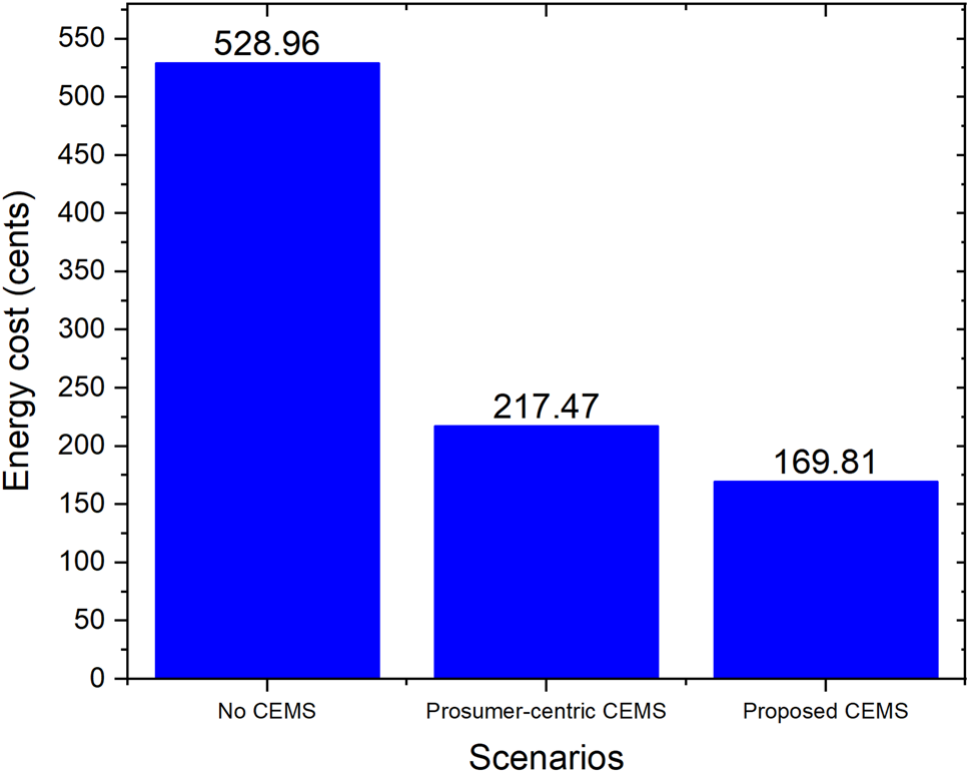
Daily energy cost of community with three CEMSs.

**Figure 7:**
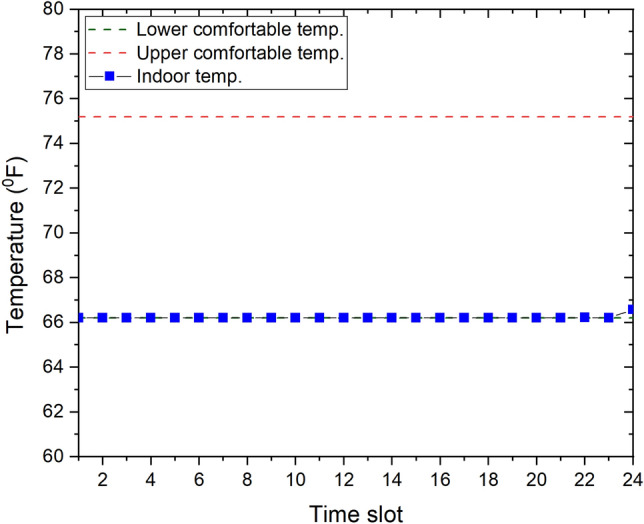
Indoor temperature during day at every home of community with thee CEMSs.

Figure 6 depicts the energy cost of our proposed community with three CEMSs for a day, whereas the indoor temperature during this day, which is the same at every home, is illustrated in Fig. 7. As indicated in these figures, the thermal comfort is satisfied at every home of the community; however, the energy cost of the community with no CEMS and the prosumer-centric CEMS are 528.96 cents and 217.47 cents, respectively, whereas the energy cost of the community with the proposed CEMS is only 169.81 cents, which is a significant decrease of $22\%$ compared to that of the community with the prosumer-centric CEMS. The community with the proposed CEMS is the best among the three scenarios in terms of the daily energy cost.

**Figure 8:**
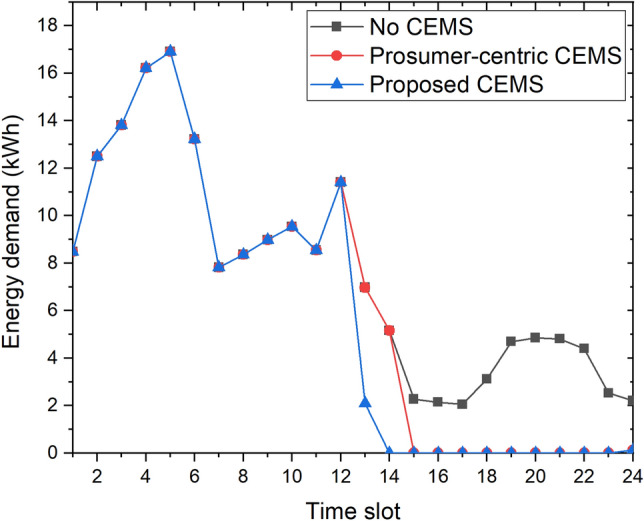
Energy demand of community from the EP with three CEMSs from EP.

**Figure 9:**
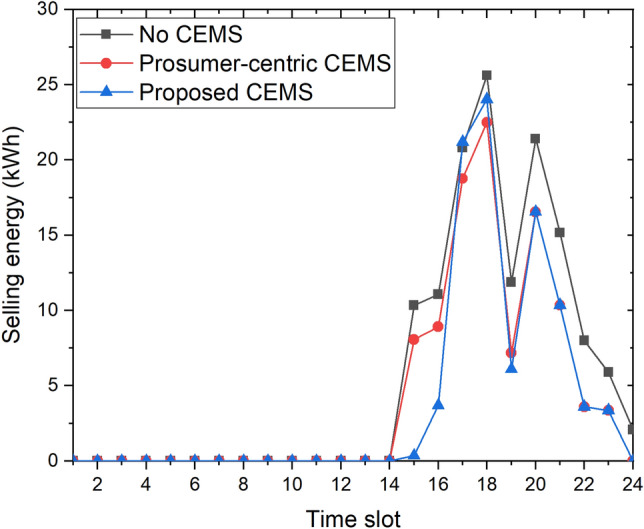
Selling energy of community with three CEMSs to EP.

The energy demand and selling energy of the community with the three CEMSs from/to the EP at each time slot are shown in Figs. 8 and 9, respectively, to gain better insight into the operation of the community. As illustrated in Fig. 8, from time slot 1 to time slot 12, the energy demand of the community with three CEMSs from the EP is almost the same because the GHI at these time slots is almost 0. However, following time slot 12, there are differences in the operations of the three scenarios when the GHI at these time slots is available. In the no-CEMS community, certain homes still require energy from the EP because they cannot buy it from other homes where the surplus energy has to be sold back to the EP. In the community with the prosumer-centric CEMS and proposed CEMS, from time slot 15 to time slot 24, the homes do not require energy from the EP because they can share energy when they have surplus energy from their PV systems or ESSs. However, at time slots 13 and 14, the energy demand of the community with the proposed CEMS is smaller than that of the community with the prosumer-centric CEMS. We have this result because, in the community with the proposed CEMS, central operation unit requires homes with DERs to continue sharing their surplus energy for other homes at these time slots. Meanwhile, in the community with prosumer-centric CEMS, homes with DERs just keep their surplus energy and do not share their surplus energy for other homes. Thus, the daily energy cost of the community with the proposed CEMS is smaller than that of the community with the prosumer-centric CEMS.

Figure 9 depicts the selling energy of the community with three CEMSs. This result is another reason that the daily energy cost of the community with the proposed CEMS is smaller than that of the others. Starting from time slot 15, the community with both the prosumer-centric CEMS and proposed CEMS sells its surplus energy to the EP. However, the community with the proposed CEMS sells more energy than the community with the prosumer-centric CEMS at high-price time slots (e.g., time slots 17 and 18). In the no-CEMS scenario, the selling energy is greater than that of two cases in these times slots. However, the energy demand of the community in the no-CEMS scenarios is also substantially larger than that of the community in the other scenarios at these time slots, as indicated in Fig. 8, and the selling price is always lower than the buying price at every time slot. Hence, the daily energy cost of the community in the no-CEMS scenario is the highest among the three scenarios.

### Computational time of community with proposed CEMS

Table 4 displays the computational time of the optimization problem of the community with the proposed CEMS in four cases: 10 homes, 50 homes, 100 homes, and 500 homes.

**Table 4:** Computational time of community with proposed CEMS.

Number of homes	10	50	100	500
Time	1 (s)	3.3 (s)	10.8 (s)	118.2 (s)

Our optimization problem is an MILP problem, which is generally a type of NP-hard problem. To the best of our knowledge, no polynomial-time algorithm is available that can be applied to solve all MILP problems. However, not every MILP is an NP-hard problem. Moreover, at present, efficient algorithms are available in which certain MILP problems can be relaxed and solved within a reasonable amount of time when they are combined with advanced mathematical solvers (e.g., CPLEX) in commercial software. In our optimization problem, the computational times for the 100-home and 500-home communities are 10.8 s and 118.2 s, respectively, in the AIMMS software. It is clear that these times are sufficiently small for building a day-ahead schedule for a community.

### Daily energy cost of each home in community with three CEMSs

**Figure 10:**
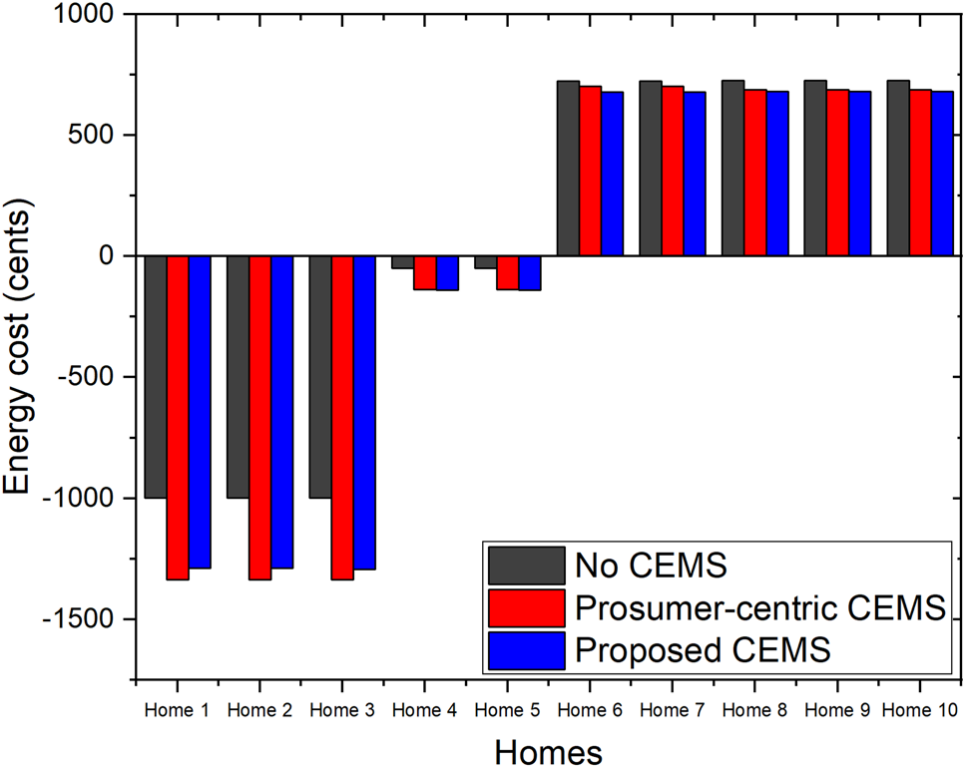
Energy cost of each home in community with three CEMSs.

The daily energy cost of each home in the community with three CEMSs is shown in Fig. 10. In this figure, the daily energy costs of the homes that have PV systems are the negative values. This means that residents of these homes receive some money because the energy that is generated by the PV system is greater than the energy demand of these homes. As the local buying/selling prices are better than the buying/selling prices proposed by the EP, this cost of each home in the community with the prosumer-centric CEMS and proposed CEMS is always better than that of each home in the community with no CEMS, where every home must directly trade its surplus energy with the EP. According to this figure, the daily benefits of the homes that have PV systems and ESSs (*Home 1 to Home 3*) in the community with the proposed CEMS are worse than those in the community with the prosumer-centric CEMS; however, the daily benefits of the remaining homes in the community with the proposed CEMS are better than those in the community with the prosumer-centric CEMS. This is because, in the community with the proposed CEMS, the objective function minimizes the overall energy cost of the community. Hence, the homes with PV systems and ESSs must sacrifice their benefits and share their benefits with the other homes to achieve the minimum overall energy cost of the community. The SS of the central operation unit schedules the DERs at the homes with PV systems and ESSs by considering not only the loads at these homes, but also the loads at other homes in the community. In contrast, in the community with the prosumer-centric CEMS, the objective function minimizes the energy cost of each home and the schedule of the DERs at homes with PV systems and ESSs that is generated based only on the loads of this home.

**Figure 11:**
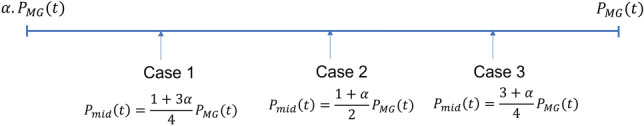
Values of $P_{mid}(t)$ considered in three cases.

A special feature of MMR pricing is that the local buying and selling prices of the community, $P_{LB}(t)$ and $P_{LS}(t)$, can be flexibly changed by adjusting the value of variable $P_{mid}(t)$. We consider the daily energy cost of the homes in the community with the proposed CEMS under different values of variable $P_{mid}(t)$, as shown in Fig. 11.

48 $$\begin{aligned} P_{mid}(t) = {\left\{ \begin{array}{ll} (1+3 \cdot \alpha )\cdot P_{MG}(t)/4 &{} \quad \text {Case 1} \\ (1+\alpha )\cdot P_{MG}(t)/2 &{} \quad \text {Case 2} \\ (3+\alpha )\cdot P_{MG}(t)/4 &{} \quad \text {Case 3} \end{array}\right. } \end{aligned}$$

**Figure 12:**
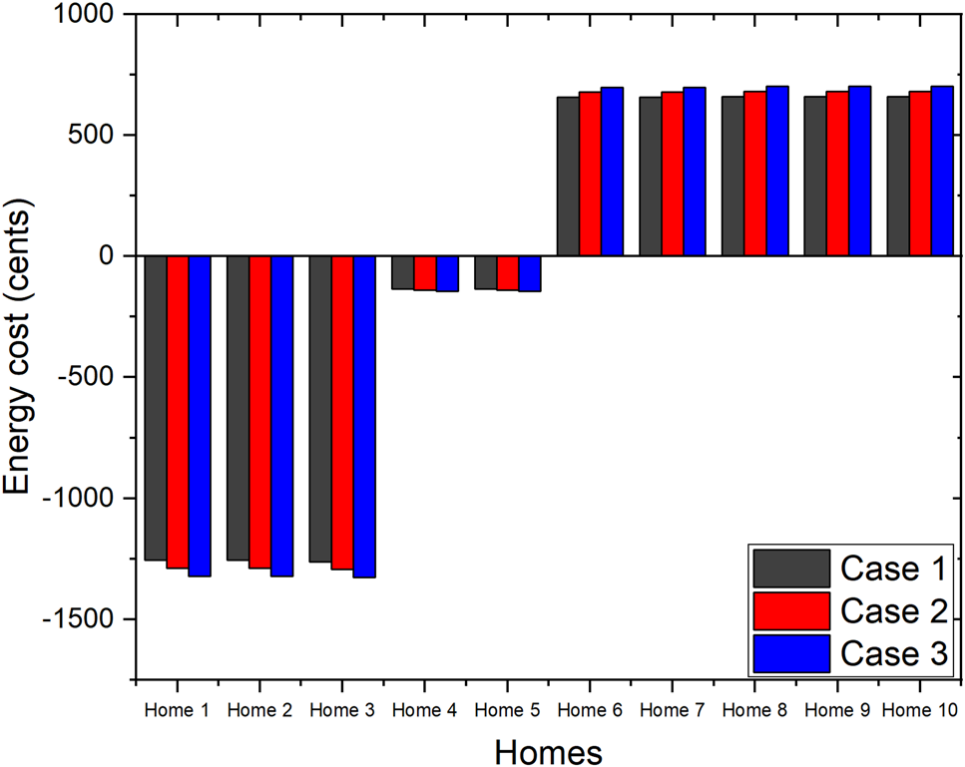
Trade-off between benefits of homes in community with our proposed CEMS under different values of $P_{mid}(t)$.

Figure 12 depicts the daily energy cost of each home in the community with the proposed CEMS under three values of variable $P_{mid}(t)$. It is clear that there is a trade-off occurs between the benefits of the homes that have and do not have a PV system in a community that applies MMR pricing. The benefits of the homes that have a PV system are increased and the benefits of the homes that do not have a PV system are decreased steadily when variable $P_{mid}(t)$ is increased.

By using this special feature, the daily benefits of the different types of homes in the community with the proposed CEMS can be flexibly increased or decreased.

## Discussion

One of main problems of a system-centric CEMS is the poor computational time for solving its optimization problem^1^. However, in this study, using advanced mathematical solvers in AIMMS software, we demonstrate that the optimization problem of a community with the proposed CEMS can be transformed and solved within a short time even for a large community with 500 homes. Moreover, compared to the prosumer-centric CEMS, the benefit that the proposed CEMS provides to the community is significant: the community with the proposed CEMS only has $78\%$ of the daily energy cost of the community with the prosumer-centric CEMS. In the proposed CEMS, a central operation unit can flexibly increase or decrease the benefits of different types of homes depending on the policy of the community by using local energy trading with MMR pricing. This is a significant advantage of CB-LEM compared to P2P-LEM. Owing to these advantages, the system-centric CEMS and CB-LEM still exhibit enormous potential for future studies.

Other important problems of the system-centric CEMS are how to protect the private information of prosumers and a single point of failure at the central operation unit of the community. However, these problems are beyond the scope of this study.

## Conclusions and future works

In this study, an optimization problem for a system-centric CEMS that supports different types of homes has been constructed and solved. Our proposed CEMS also supports local energy trading between homes using MMR pricing. For the comparison and validation of performance between our proposed model and existing models, we also built two different CEMSs: a prosumer-centric CEMS and no CEMS. These CEMSs were applied to the same community in Detroit city, Michigan, USA where real data of Detroit environment was used for simulations. The simulation results showed that, in this community, the daily energy costs of a prosumer-centric CEMS and no CEMS were 217.47 cents and 528.96 cents, respectively. Whereas, the daily energy cost of the proposed CEMS was smaller than other CEMSs with only 169.81 cents, a significant decrease of $22\%$ compared with prosumer-centric CEMS. The number of homes of the community was varied in our simulations for evaluation of the computational time of the proposed CEMS. The computational time of the proposed CEMS for a 500-home community was only 118.2 s, which is a sufficiently short time for day-ahead scheduling. It means that the proposed CEMS can be widely applied to many existing communities. Furthermore, with local energy trading using MMR pricing, the benefits of several homes could be changed flexibly by adjusting the value of variable $P_{mid}(t)$. This special feature of MMR pricing helps the central operation unit of the community to change its energy management policy easily.

The security for a system-centric CEMS may be a potential field for future studies. Another research direction is to develop a cooperative strategy for many communities, whereby a community can buy or sell energy from another community.

## Data Availability

The day-ahead GHI used in this article is from a photovoltaic geographical information system database^35^. The day-ahead outside temperature of a day in Detroit city is from the Kaggle website^36^. The day-ahead hourly prices of the EP in Detroit city, which were extracted from the Pecan Street database^37^. The datasets used and/or analysed during the current study available from the corresponding author on reasonable request.

## Abbreviations

ADMM: Alternating direction method of multipliers
CB-LEM: Community-based local energy market
CEMS: Community energy management system
DER: Distributed energy resource
EP: Electricity provider
ESS: Energy storage system
HVAC: Heating, ventilation, and air conditioning
LEC: Local energy community
LEM: Local energy market
MILP: Mixed integer linear programming
MMR: Mid-market rate
P2P-LEM: Peer-to-peer local energy market
PSO: Particle swarm optimization
PV: Photovoltaics
RES: Renewable energy system
SS: Smart scheduler
UDDSR: User-dominated demand side response

## Indices and set

*i*,*N*: Index and number of homes of the community
*t*,*T*,$\Delta t$: Index, number of time slots, and duration of a time slot in a day

## Parameters and constants

$\alpha \cdot P_{MG}(t)$: Day-ahead selling price at which community sells electricity to EP in time slot *t* (cents/kWh)
$\eta$: Thermal conversion efficiency
$$\eta ^{ESS}$$: ESS efficiency
$\eta ^{RES}$: Solar conversion efficiency of PV system
$$\varepsilon$$: System inertia
*A*: Overall thermal conductivity
$Ch_{i,rate}$: Maximum charge rate of ESS of home *i* (kW)
$Dh_{i,rate}$: Maximum discharge rate of ESS of home *i* (kW)
$E_{i,Fix}(t)$: Fixed load of home *i* in time slot *t* (kWh)
$E_{i,max}$: Energy peak of home *i* (capacity of power cable of home *i*) (kWh)
$E_{max}$: Energy peak (capacity of power cable of community) (kWh)
$EL_{i,0}$: Initial energy level of ESS of home *i* (kWh)
$EL_{i,max}$: Maximum energy level of ESS of home *i* (kWh)
$EL_{i,min}$: Minimum energy level of ESS of home *i* (kWh)
$GHI_i(t)$: Global horizontal irradiation at home *i* in time slot *t* ($\text{kW}/\text{m}^2$)
*M*: Very large value
$P_{max}$: Rating power of HVAC system (kW)
$P_{MG}(t)$: Day-ahead buying price at which community buys electricity from EP in time slot *t* (cents/kWh)
$P_{mid}(t)$: Price with value between $\alpha \cdot P_{MG}(t)$ and $P_{MG}(t)$ in time slot *t* (cents/kWh)
$S_i$: Total area of solar panels of home *i* ($\text{m}^2$)
$T^{out}(t)$: Outdoor temperature in time slot *t* ($^\text {o}$F)
$T_{min}$, $T_{max}$: Minimum and maximum comfortable indoor temperatures ($^\text {o}$F)

## Variables

$C_{Com}(t)$: Energy cost of community in time slot *t* (cents)
$C_{i}$: Daily energy cost of home *i* (cents)
$E_{Com}(t)$: Total buying/selling energy of community from/to EP in time slot *t* (kWh)
$E_{i,Com}(t)$: Total buying/selling energy of home *i* from/to community in time slot *t* (kWh)
$E_{i,Com}^{buy}(t)$: Total buying energy of home *i* from community in time slot *t* (kWh)
$E_{i,Com}^{charge}(t)$: Energy from community used for storage in ESS of home *i* in time slot *t* (kWh)
$E_{i,Com}^{load}(t)$: Energy from community used for appliances of home *i* in time slot *t* (kWh)
$E_{i,Com}^{sell}(t)$: Total selling energy of home *i* to community in time slot *t* (kWh)
$E_{i,ESS}^{Level}(t)$: Energy level of ESS of home *i* after time slot *t* (kWh)
$E_{i,ESS}^{load}(t)$: Energy from ESS of home *i* used for appliances in time slot *t* (kWh)
$E_{i,ESS}^{sell}(t)$: Energy from ESS of home *i* used for selling to community in time slot *t* (kWh)
$E_{i,HVAC}(t)$: Input energy of HVAC system of home *i* in time slot *t* (kWh)
$E_{i,RES}(t)$: Output energy from PV system of home *i* in time slot *t* (kWh)
$E_{i,RES}^{charge}(t)$: Energy from RES of home *i* used for storage in ESS in time slot *t* (kWh)
$E_{i,RES}^{load}(t)$: Energy from RES of home *i* used for appliances in time slot *t* (kWh)
$E_{i,RES}^{sell}(t)$: Energy from RES of home *i* used for selling to community in time slot *t* (kWh)
$mode_{i,ESS}(t)$: Binary variable indicating charging/discharging status of ESS of home *i* in time slot *t*
$mode_{i,home}(t)$: Binary variable indicating buying/selling status of home *i* in time slot *t*
*P*(*t*): Price of the community with proposed CEMS buying/selling from/to EP in time slot *t* (cents/kWh)
$P_{i}(t)$: Price of home *i* within the community with prosumer-centric CEMS in time slot *t* (cents/kWh)
$p_{i}(t)$: Input power of HVAC system of home *i* in time slot *t* (kW)
$P_{LB}(t)$: Local buying price proposed by central operation unit in time slot *t* (cents/kWh)
$P_{LS}(t)$: Local selling price proposed by central operation unit in time slot *t* (cents/kWh)
$P_{L}(t)$: Local price proposed by central operation unit in time slot *t* (cents/kWh)
*s*(*t*): Binary variable indicating buying/selling status of community in time slot *t*
$T_{i}^{in}(t)$: Indoor temperature of home *i* in time slot *t* ($^\text {o}$F)
